# An *in situ* and *in vitro* investigation of cytoplasmic TDP-43 inclusions reveals the absence of a clear amyloid signature

**DOI:** 10.1080/07853890.2022.2148734

**Published:** 2022-12-10

**Authors:** Roberta Cascella, Martina Banchelli, Seyyed Abolghasem Ghadami, Diletta Ami, Maria Cristina Gagliani, Alessandra Bigi, Tommaso Staderini, Davide Tampellini, Katia Cortese, Cristina Cecchi, Antonino Natalello, Hadi Adibi, Paolo Matteini, Fabrizio Chiti

**Affiliations:** a Department of Experimental and Clinical Biomedical Sciences “Mario Serio”, University of Florence, Florence, Italy; b Institute of Applied Physics “Nello Carrara”, National Research Council, Sesto Fiorentino, Italy; c Department of Biotechnology, Faculty of Biological Sciences, Alzahra University, Tehran, Iran; d Department of Biotechnologies and Biosciences, University of Milano-Bicocca, Milan, Italy; e Milan Center of Neuroscience (NeuroMI), Milan, Italy; f Cellular Electron Microscopy Laboratory, Department of Experimental Medicine, University of Genova, Genoa, Italy; g U 1195 INSERM-Université Paris-Saclay, Paris, France; h Institut Professeur Baulieu, Paris, France; i Pharmaceutical Sciences Research Center, Health Institute, Kermanshah University of Medical Sciences, Kermanshah, Iran

**Keywords:** Motor neuron disease, MND, Lou Gehrig’s disease, amyotrophic lateral sclerosis, frontotemporal lobar degeneration, ALS, FTLD, TDP-43 filaments

## Abstract

**Introduction:** Several neurodegenerative conditions are associated with a common histopathology within neurons of the central nervous system, consisting of the deposition of cytoplasmic inclusions of TAR DNA-binding protein 43 (TDP-43). Such inclusions have variably been described as morphologically and molecularly ordered aggregates having amyloid properties, as filaments without the cross-β-structure and dye binding specific for amyloid, or as amorphous aggregates with no defined structure and fibrillar morphology.

**Aims and Methods:** Here we have expressed human full-length TDP-43 in neuroblastoma x spinal cord 34 (NSC-34) cells to investigate the morphological, structural, and tinctorial properties of TDP-43 inclusions *in situ*. We have used last-generation amyloid diagnostic probes able to cross the cell membrane and detect amyloid in the cytoplasm and have adopted Raman and Fourier transform infrared microspectroscopies to study *in situ* the secondary structure of the TDP-43 protein in the inclusions. We have then used transmission electron microscopy to study the morphology of the TDP-43 inclusions.

**Results:** The results show the absence of amyloid dye binding, the lack of an enrichment of cross-β structure in the inclusions, and of a fibrillar texture in the round inclusions. The aggregates formed *in vitro* from the purified protein under conditions in which it is initially native also lack all these characteristics, ruling out a clear amyloid-like signature.

**Conclusions:** These findings indicate a low propensity of TDP-43 to form amyloid fibrils and even non-amyloid filaments, under conditions in which the protein is initially native and undergoes its typical nucleus-to-cell mislocalization. It cannot be excluded that filaments emerge on the long time scale from such inclusions, but the high propensity of the protein to form initially other types of inclusions appear to be an essential characteristic of TDP-43 proteinopathies.

**KEY MESSAGES**
- Cytoplasmic inclusions of TDP-43 formed in NSC-34 cells do not stain with amyloid-diagnostic dyes, are not enriched with cross-β structure, and do not show a fibrillar morphology.
- TDP-43 assemblies formed *in vitro* from pure TDP-43 do not have any hallmarks of amyloid.

## Introduction

Frontotemporal lobar degeneration with ubiquitin-positive inclusions (FTLD-U or FTLD-TDP) and amyotrophic lateral sclerosis (ALS) are two highly widespread neurodegenerative conditions associated in most cases with a common histopathology within neurons of the central nervous system, consisting in neuronal cytoplasmic inclusions (NCIs) of TAR DNA-binding protein 43 (TDP-43) in these cells [1–6]. In ALS, TDP-43 inclusions accumulate in the motoneurons of the primary motor cortex, corticospinal tracts, brainstem, spinal cord, glial cells, and also in the neurons of the hippocampus and frontotemporal cortex in a subset of patients [2,5,7]. In FTLD-U the NCIs are mainly present in the frontal and temporal cortices and in the hippocampus and their glial cells [2,8]. TDP-43 inclusions are also frequently found in the brains of Alzheimer’s disease [9,10], Parkinson’s disease [9,11], Huntington’s disease [12], and other neurodegenerative conditions [13], although in this case they are thought to represent a secondary effect.

Formation of TDP-43 NCIs is associated with the mislocalization of the protein from the nucleus, where it normally resides and plays its function, to the cytoplasm [1–4]. It is likely that newly formed inclusions in the cytoplasm recruit functional TDP-43, which therefore has a spatial shift from its native compartment to the cytoplasm [13]. In this cellular district, it forms deleterious aggregates causing cell dysfunction *per se*, where TDP-43 is hyperphosphorylated, polyubiquitinated and partially proteolyzed [1–4,6,14,15]. The combination of these major events in the nucleus and cytoplasm underlines that TDP-43 proteinopathies originate from a combination of loss-of-function (LOF) and gain-of-function (GOF) mechanisms [16–19].

TDP-43 is a complex multi-domain protein of 414 amino acid residues. Its discovery in the inclusions of diseases that are as widespread as ALS and FTLD-U dates back to 2006 [1,2], i.e. very recently relative to aggregated proteins of similarly widespread neurodegenerative conditions, witnessing the difficulty to characterize the histological, structural, and morphological characteristics of TDP-43 aggregates. Its purification as a full-length and native protein to a high yield, in the absence of large solubilizing tags, through reproducible and routinely used procedures, was attained even more recently, starting from 2019, with only occasional attempts until then [20]. This double technological limitation has hindered the elucidation of its aggregation process *in cell* and *in vitro* and of the structural/morphological characterization of the resulting assemblies. In particular, it has long been debated whether TDP-43 NCIs have the characteristic order and cross-β structure typical of amyloid fibrils or rather another type of molecular architecture. Protein aggregates need to possess three main hallmarks to be classified as amyloid: (i) a fibrillar morphology with typical diameters of the individual fibrils of *ca.* 7–13 nm, (ii) binding to amyloid-diagnostic dyes, (iii) a cross-β secondary structure [21–23]. The identification of these three hallmarks in TDP-43 aggregates has so far remained elusive, both *in vitro* and *in vivo*.

Under conventional immunohistochemistry, the inclusions appear either skein-like or compact and round [1–5,24,25]. Round inclusions range from 1 to 25 µm in diameter in neurons, although smaller species are likely to be present and escape detection due to microscopical resolution limitations [3,5,24,25]. Skein-like inclusions are *ca*. 0.5–1.0 µm in diameter and up to 15 µm in length [3,5,24,25]. Histopathologists have repeatedly reported transmission electron microscopy (TEM) images of 10–20 nm wide filaments containing TDP-43 in spinal cord sections of ALS cases and brain specimens of FTLD-U patients [10,14,26–30], in the absence of binding to amyloid diagnostic dyes [31–35]. A report exists, however, indicating the presence of widespread thioflavin S (ThS) staining in ALS spinal cords and FTLD-U brains, suggesting rather an amyloid structure [36]. In another report, it was shown that TDP-43 inclusions of ALS cases may bind ThS, but such features were found only in a small fraction of skein-like inclusions of the spinal cord, with amyloid characteristics being absent in most spinal cord skeins and absent altogether in round TDP-43 inclusions of the spinal cord and in all brain inclusions [28].

A major breakthrough is offered by the recent structural determination, by cryo-electron microscopy (cryo-EM), of the protease-resistant portion of filaments extracted from the frontal and motor cortex of two cases with a history of ALS associated with FTLD-U [30]. In the filaments, residues 282–360 from TDP-43 molecules stack on each other in a parallel, in-register fashion at 4.8 Å distance and 1.4° angles to form a right-handed double-spiral fold. Each molecule forms 10 very short β-strands alternated by large stretched of turns and the β-sheets do not stack on each other and do not form, consequently, the cross-β structure typical of amyloid fibrils [30]. In addition, the smooth profilament surface does not present deep and non-polar grooves to bind amyloid-diagnostic dyes, offering an explanation for the reason why dye binding and cross-β structure remain undetected in biological TDP-43 NCIs [30]. Even more recently it was found, by three independent groups concomitantly, that *bona fide* amyloid fibrils by the previously undetected endosomal and lysosomal TMEM106B C-terminal domain (CTD) are often found in aged brains of several patients suffering from different neurodegenerative diseases, including FTLD-U and ALS cases [37–39]. Therefore, the positivity to amyloid diagnostic dyes found in a few ALS and FTLD-U cases might arise from TMEM106B CTD fibrils.

In this work, we have expressed human, full-length, tag-free TDP-43 in NSC-34 cells and have tried to investigate *in situ* the cytoplasmic TDP-43 inclusions with recently developed methods that allow structural information to be obtained directly *in cell*, such as their secondary structure and amyloid-diagnostic dye binding. We have also studied the structure of the assemblies formed *in vitro* from the same purified tag-free native protein, confirming data obtained *in cell*. In both cases, we did not find any of the typical hallmarks of amyloid and we could not even identify a filamentous texture within the inclusions formed in the cells or in the test-tubes, indicating that the protein has a high propensity to form other types of assemblies, at least in the initial steps of the aggregation process. Unlike the work performed on human biological specimens, in which the TDP-43 NCIs are often ‘contaminated’ by TMEM106B CTD amyloid fibrils and are not routinely available, those forming in our two conditions form on the time scale of a few days and in the absence of TMEM106B CTD aggregates, allowing the investigation of the initial TDP-43 inclusions to be studied more promptly.

## Materials and methods

### Cell cultures

Murine NSC-34 is a hybrid motoneuron/neuroblastoma cell line that retains the ability to proliferate and express several motor neuron characteristics [40]. NSC-34 cells were routinely maintained in DMEM, with 5% fetal bovine serum (FBS), 1 mM glutamine, 1.0% sodium pyruvate, and antibiotics (cell medium), in a 5% CO_2_ humidified atmosphere at 37 °C and grown until they reached 80% confluence, for a maximum of 20 passages [18,41].

In one experiment, primary neurons were obtained from male heterozygous PS19 transgenic mice [42], harboring the human tau gene with the P301S mutation for a familial form of frontotemporal dementia (Jackson Laboratory), crossed with female B6C3F1/N wild-type mice. Primary neuronal cultures were prepared from hippocampi and cortices of E15 mouse embryos, as described [43], plated on poly-D-lysine (Sigma-Aldrich) coated 2-well Chamber Slide with removable wells (Thermo Fisher Scientific) and maintained in neurobasal medium containing penicillin/streptomycin (Thermo Fisher Scientific), B-27 supplement (Thermo Fisher Scientific), and glutamine (Sigma-Aldrich). Each culture was genotyped by PCR using specific primers for human tau and the protocol provided by Jackson Laboratory (https://www.jax.org/Protocol?stockNumber=008169&protocolID=34005). Primary neurons were used at 14 days *in vitro* (DIV) for all experiments. All procedures were in strict compliance with the recommendations of the EU Directive 2010/63/EU for animal experiments and were approved by the Ministry of National Education, Higher Education and Research (France).

### Transient transfection

Overexpression of TDP-43 was carried out using the pCI-neo plasmid expressing human TDP-43 (kindly provided by E. Buratti, Italy), as previously reported [18]. NSC-34 cells were plated in 12-well plates containing coverslips at 75,000 cells/well density. Twenty-four hours after plating, the cells were washed with PBS and transfected using Lipofectamine 3000 (Life Technologies), according to the manufacturer’s instructions, with 20 µg of plasmid, 3.5 µl of Lipofectamine 3000 Reagent, 5 µl of 5 mg/l transferrin, and 2.5 µl of P3000 reagent in DMEM for 3 h in a 5% CO_2_ humidified atmosphere at 37 °C. After 3 h, this medium was replaced with fresh complete cell medium, the cells were incubated for 40 h and then STED images were acquired, as reported below.

### STED microscopy

STED *xyz* images (*z*-stacks acquired along *x*/*y*/*z* axes) of NSC-34 cells were acquired in bidirectional mode using an SP8 STED 3× confocal microscope (Leica Microsystems), as previously reported [44]. Total TDP-43 was monitored using 1:300 rabbit polyclonal anti-TDP-43 antibody (Proteintech), in PBS plus 1% FBS, for 60 min at 37 °C, and then with 1:500 Alexa Fluor 568 (or Alexa Fluor 514) conjugated secondary antibodies (Life Technologies), in PBS plus 1% FBS, for 60 min at 37 °C. Fluoromount-G™ (Fisher Scientific) was used as the mounting medium. Alexa Fluor 568 and Alexa Fluor 514 were excited with a 561 nm and 510 nm tuned white light laser (WLL) and emission was collected at 580–620 and 532–551 nm, respectively. The cell membrane was labelled with wheat germ agglutinin (WGA) Tetramethylrhodamine (TMR) Conjugate (Life Technologies) for a 3D reconstruction of the whole cell. TMR was excited with a 550 nm-tuned WLL and emission was collected at 564–599 nm. Frame sequential acquisition was applied to avoid fluorescence overlap. A gating of 0.3–6 ns was applied to avoid the collection of reflection and autofluorescence. Images were acquired with Leica HC PL APO CS2 100×/1.40 oil STED White objective and gated pulsed-STED was applied. Collected images were de-convolved with Huygens Professional software version 18.04 (Scientific Volume Imaging B.V.) and analyzed with Leica Application Suite X (LAS X) software (Leica Microsystems) to generate 3D reconstructions. Z-series stacks were obtained from 5 μm cell slices. Images were collected at 0.1 μm intervals.

### Staining with amyloid-diagnostic dyes

NSC-34 cells were plated and transfected with either vehicle or the pCI-neo plasmid expressing TDP-43 and analyzed after 40 h, as reported above. NSC-34 cells were also transfected with 0.15 µM native BSA or 2 µM Aβ_42_ fibrils, prepared by dissolving the Aβ_42_ peptide in DMSO to 5 mM, diluting in 10 mM HCl to a final concentration of 100 μM and incubating the resulting sample at 37 °C without agitation for 1 day, as previously described [45]. The transfection with BSA or Aβ_42_ fibrils was carried out using the PULSin protein delivery reagent (Polyplus-transfection), in the presence of 100 µl of 20 mM Hepes Buffer and 4 µl of PLUSin reagent in cell culture medium without FBS for 2 h, and the analysis was performed immediately. After transfection, cells were washed with PBS, fixed in 2% (*w/v*) buffered paraformaldehyde for 10 min at 20 °C, and permeabilized with a 0.5% (*v/v*) Triton X-100 solution for 5 min. The presence of amyloid structures was then monitored by using the amyloid-diagnostic dyes, such as 1:300 diluted Amitracker 630 [46] (Ebba Biotech), or 130 µM compounds **3** [47] and **4j** [48] for 1 h at 37 °C. Full names of compounds **3** and **4j** are (*E*)-6-hydroxy-2-(2-hydroxy-3-methoxybenzylidene)benzofuran-3(2*H*)-one and 5-(2-chlorophenyl)-8-(dimethylamino)-2-thioxo-1,2,3,5-tetrahydro-4H-chromeno[2,3-*d*]pyrimidin-4-one, respectively. Coverslips stained with Amitracker 630 were also incubated in PBS containing DAPI for 15 min at 20 °C to detect cellular nuclei. Fluorescence emission was detected after excitation at 405 nm and 514 nm for coverslips stained with DAPI and Amitracker 630, and 488 nm for those labelled with compounds **3** and **4j**, respectively, by the TCS SP8 scanning confocal microscopy system (Leica Microsystems) equipped with an argon laser source, using a Leica Plan Apo 63× oil immersion objective. The confocal microscope was set at optimal acquisition conditions, e.g. pinhole diameters, detector gain, and laser powers. Settings were maintained constant for each analysis.

### Raman spectroscopy

NSC-34 cells were plated in 12-well plates containing coverslips at 75,000 cells/well density and transfected with 80 µg of wtTDP43tdTOMATOHA plasmid (Addgene) coding for human TDP-43 C-terminally fused to the fluorescent tdTOMATO protein, using 3.5 µl of Lipofectamine 3000 Reagent, 5 µl of 5 mg/l transferrin, and 2.5 µl of P3000 reagent in DMEM for 3 h in a 5% CO_2_ humidified atmosphere at 37 °C. NSC-34 cells were also transfected with vehicle (control cells). After 40 h, they were then pelleted (200 g for 6 min) and washed twice with PBS between successive centrifugations. For a single Raman measurement, 2 μl of the cell pellet was drop-casted onto a gold mirror support (ME1S-M01; Thorlabs, Inc.). Raman experiments were carried out on a micro-Horiba Xplora coupled to a 532 nm and a 785 nm wavelength laser for the excitation. The microspectrometer used a 1200 grooves mm-1 grating with a confocal microscope in backscattering geometry and a 2 D-CCD camera. The backscattered light was collected by a 100× microscope objective with 0.9 NA, which generated *a* ≈ 2 μm large laser beam waist. An integration time of 5 s and a laser power value of 1 mW on the sample were employed for Raman measurements on the cells. For each sample at least five individual cells were inspected, each in three areas enriched with fluorescent tdTOMATO protein, while carefully avoiding cell dehydration. The Raman spectra of each sample were averaged and baseline-corrected. To monitor only the cells overexpressing tdTOMATO protein, fluorescence emission in the 550–700 nm range was measured in the areas selected for Raman experiments, by exciting at 532 nm with a 10× (0.25 NA) objective.

### Fitting analysis of Raman spectra

Each Raman spectrum from 1500 to 1800 cm^−1^, containing the amide I band, was isolated and analyzed with a fitting procedure using the sum of five Lorentzian functions, one for the 1580–1590 cm^−1^ peak, one for the 1610–1625 cm^−1^ peak and the remaining three for the α-helical component (1655–1665 cm^−1^), β-sheet component (1665–1675 cm^−1^), and unstructured component (>1675 cm^−1^):

(1) $$y=\;\sum_{i=1}^{5}\{A_{i}(\frac{1}{\pi })(\frac{a_{i}}{2})/[(x-x_{0i}{)}^{2}+(\frac{a_{i}}{2\;}{)}^{2}]\}$$

where *A_i_* and *a_i_* are parameters related to the height and width of the Lorentzian function *i*, respectively, and *x_0i_* is its wavenumber of maximum intensity (peak). The 15 parameters of the five Lorentzian functions (*A_i_*, *a_i_*, *x_0i_* for all five functions) were left free to float in the fitting procedure simultaneously, but their *x_0i_* values were restrained within their corresponding 10 cm^−1^ range. Moreover, since the α-helical, β-sheet, and unstructured components are known to be 36, 22, and 42% in the human and mammalian proteome [49,50], their relative areas were restrained to float within *a* ± 50% range around these percentage values.

### FTIR microspectroscopy

NSC-34 cells were plated in 12-well plates containing coverslips at 75,000 cells/well density and transfected with either vehicle or the pCI-neo plasmid expressing TDP-43. NSC-34 cells were also transfected with vehicle (control cells). After 40 h, they were then pelleted (200 g for 6 min) and washed twice with 0.9% NaCl between successive centrifugations and finally deposited onto a BaF_2_ window and dried at room temperature for 30 min. FTIR spectra were then measured in transmission mode by the infrared microscope Varian 610-IR coupled to the Varian 670-IR FTIR spectrometer (Varian Australia Pty Ltd), equipped with a mercury cadmium telluride nitrogen-cooled detector, using the following settings: variable microscope aperture set at ∼100 × 100 μm, spectral resolution 2.0 cm^−1^, scan speed 25 kHz, 512 scan co-additions, and triangular apodization. The absorption spectra were normalized at the amide I band area, for comparison, and the second derivative analysis was performed, after a binomial 13-point smoothing of the normalized absorption spectra, by the Savitzky-Golay method (3rd polynomial, window of nine points), using the GRAMS/32 software (Galactic Ind. Corp.).

### TEM

Primary neuronal cultures from PS19 mice expressing P301S mutant human tau and cultured NSC-34 cells transfected with vehicle or human TDP-43 expressing plasmid were fixed, 40 h after transfection, with 2.5% glutaraldehyde (Electron Microscopy Science) in 0.1 M cacodylate buffer for 1 h at room temperature, post-fixed in 1% OsO_4_ for 1 h, 1% tannic acid for 30 min and *en bloc* stained with 1% uranyl acetate for another hour. Then samples were dehydrated through a graded ethanol series and flat embedded in epoxy resin (Poly-Bed; Polysciences, Inc.) for 24 h at 60 °C. Ultrathin sectioning (50 nm) was performed with Leica ultramicrotomes (Reichert Ultracut, Leica microsystems). Flat-embedded cells were cut parallel to the substrate and counterstained with 5% uranyl acetate in 50% ethanol. Ultrastructural analysis was performed with a Hitachi 7800 120 kV electron microscope (Hitachi) operating at 100 kV using a Megaview G3 digital camera and Radius software (EMSIS). Electron micrographs were taken using the Multiple Image Alignment (MIA) montage and screenshot tools.

### TDP-43 purification

Human full-length TDP-43 was recombinantly expressed in *Escherichia coli* BL21(DE3) cells and purified as previously described [20]. In brief, inclusion bodies (IBs) containing TDP-43 were washed and dissolved in a buffer containing 8 M urea. Then, the sample was subjected to affinity chromatography and the eluted protein was refolded in a specific refolding buffer and purified with size exclusion chromatography and anion exchange chromatography, as previously described [20]. The last chromatographic step elution was carried out using 50 mM Hepes, 1 M NaCl, 2 mM Lauryldimethylamine oxide (LDAO), 0.25% (w/v) Octyl-β-D-Glucopyranoside (OG), 0.1% (w/v) PEG3350, pH 8.0. The protein sample was concentrated using Amicon Ultra centrifugal filter units with 10 kDa molecular weight cut-off to a final concentration of *ca.* 1 mg ml^−1^ and stored at −20 °C. Protein concentration was determined by optical absorbance spectroscopy using a molar extinction coefficient at 280 nm (*ε*_280_) of 46,410 M^−1 ^cm^−1^. The final purified protein had the sequence stretch MHHHHHHSSGVDLGTENLYFQS at the N-terminus before Met1, contained 436 residues, and had a molecular weight of 47,292.53 Da.

### Confocal and STED microscopy

The purified TDP-43 sample was thawed and large aggregates were spun down at 18,000 g, 4 °C for 15 min. TDP-43 was labelled with Tetramethylrhodamine-5-maleimide (TMR) (Invitrogen) in a 1:10 ratio (dye:protein) and then subjected to desalting through a Sephadex G15 resin column (Pharmacia Fine Chemicals). Protein concentration was monitored with NanoDrop One (ThermoFisher Scientific). Aggregation was induced by diluting the protein down to 5 μM into 20 mM acetate buffer, 150 mM NaCl, 5% (w/v) PEG8000, 2 mM TCEP, pH 5.0, to reach a final pH of 6.0, and incubating under shaking at 560 rpm on a TS-100 Thermo Shaker (Kisker) at 25 °C for 4 or 10 days. Then, 40 µl of the protein aggregation sample was deposited onto an 8-well Chamber Slide and TMR was excited at 550 nm, and emission was collected at 564–599 nm. Confocal and STED microscopy images were acquired by the SP8 confocal microscope described above, equipped with the Leica HC PL APO CS2 100×/1.40 oil STED White objective and the HyD (hybrid detector). A series of optical sections (z-stacks) was taken through the aggregate’s depth; images were then de-convolved with Huygens Professional software version 18.04 (Scientific Volume Imaging B.V.), and the maximum intensity projection of confocal z-stacks was obtained by superimposition. The Leica Application Suite X (LAS X) software equipped with the 3D Projection Tool (Leica Microsystems) was used to generate depth coding profiles of 3D reconstructions from de-convolved images and movies from the z-stacks.

### Thioflavin T fluorescence

TDP-43 aggregates were prepared as described in the previous subsection, with the addition of 25 μM Thioflavin T (ThT). The resulting fluorescence was recorded from 450 to 600 nm (excitation 440 nm) at 25 °C using a Cary Eclipse Fluorescence Spectrophotometer (Agilent). A small volume 3 × 3 mm quartz cell was used and the blank was subtracted from the spectrum. Other details are as previously described [51].

### Far-UV CD

TDP-43 aggregates were prepared as described above. The far-UV circular dichroism (CD) spectrum was collected over the 200–260 nm wavelength range at 25 °C using a Jasco J-810 Spectropolarimeter, averaged from 8 scans, blank-subtracted and normalized to mean residue ellipticity. A quartz 1 mm pathlength cuvette was used. Other details are as previously described [51].

### Statistical analysis

Data were expressed as means ± SEM.

## Results

### Overexpression of full length TDP-43 leads to formation of cytoplasmic inclusions

**Figure 1.**
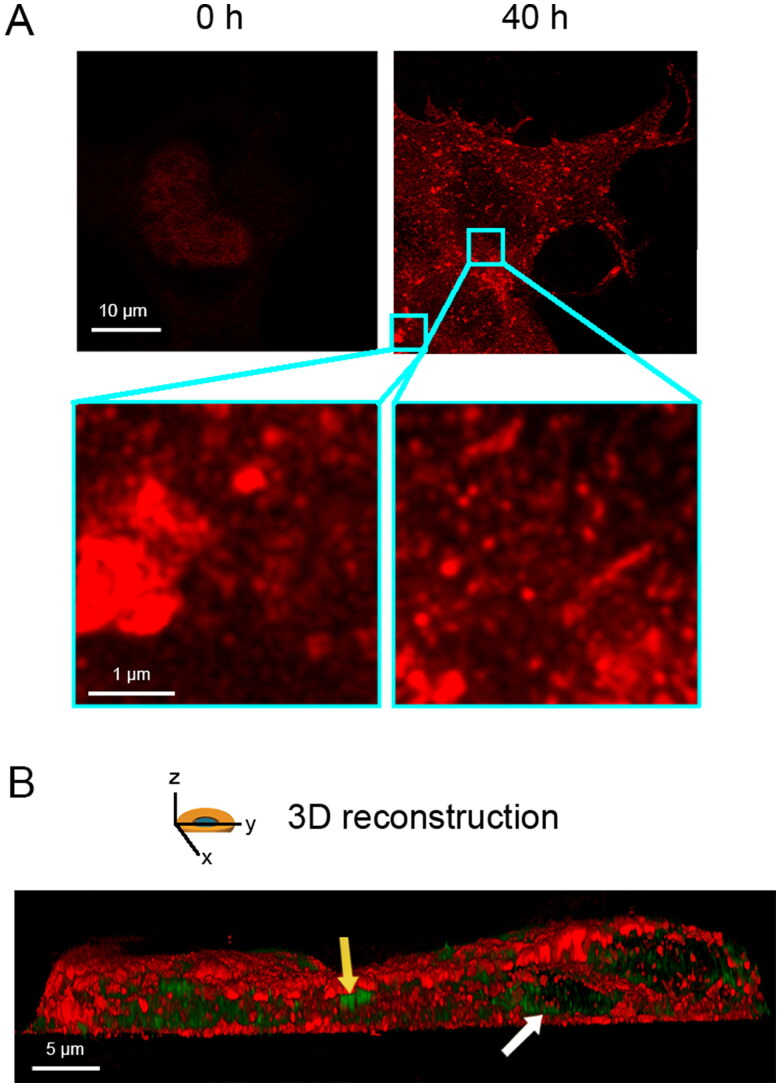
Overexpression of full length TDP-43 leads to nuclear depletion of endogenous TDP-43 and formation of TDP-43 cytoplasmic inclusions. (A) Representative STED microscopy images of NSC-34 cells (*n* = 3) transfected with 20 µg of pCI-neo plasmid expressing human TDP-43 and analyzed immediately after and 40 h after transfection. Red fluorescence: total TDP-43 (endogenous and exogenous). Higher magnifications show round (left) and elongated (right) TDP-43 inclusions. (B) 3D reconstruction of the *z*-stack analysis (5-μm-thick slices) of the specimens shown in (A). A NSC-34 cell was virtually dissected on the *zy* plane to show more clearly the intracellular TDP-43 inclusions. Red and green fluorescence: cell membrane (WGA) and exogenous (human) TDP-43, respectively. Yellow and white arrows: round and elongated inclusions, respectively.

We transiently transfected human full-length TDP-43 in NSC-34 cells, which is a motoneuron/neuroblastoma hybrid cell line that expresses several motor neuron features [40]. To this purpose, we used 20 µg of a pCI-neo plasmid coding for TDP-43 and monitored the overexpression of the protein immediately after transfection (0 h) and 40 h later with stimulated emission depletion (STED) super-resolution microscopy (Figure 1(A)). At 0 h TDP-43 was only in the nucleus, as expected, whereas at 40 h transfected cells showed both nuclear depletion and cytoplasmic deposition of TDP-43 in the form of both round and elongated inclusions (Figure 1(A), zoom), which is better visualized in a 3D reconstruction of the whole cell (Figure 1(B)). Using the same cellular and expression system, cytoplasmic TDP-43 was found in a previous report to be phosphorylated, ubiquitinated, and partially proteolyzed [18], thus recapitulating the biochemical and histopathological features observed *in vivo* under pathological conditions [1–4,6,14,15].

### Cytoplasmic inclusions of TDP-43 do not stain with amyloid-diagnostic dyes

**Figure 2.**
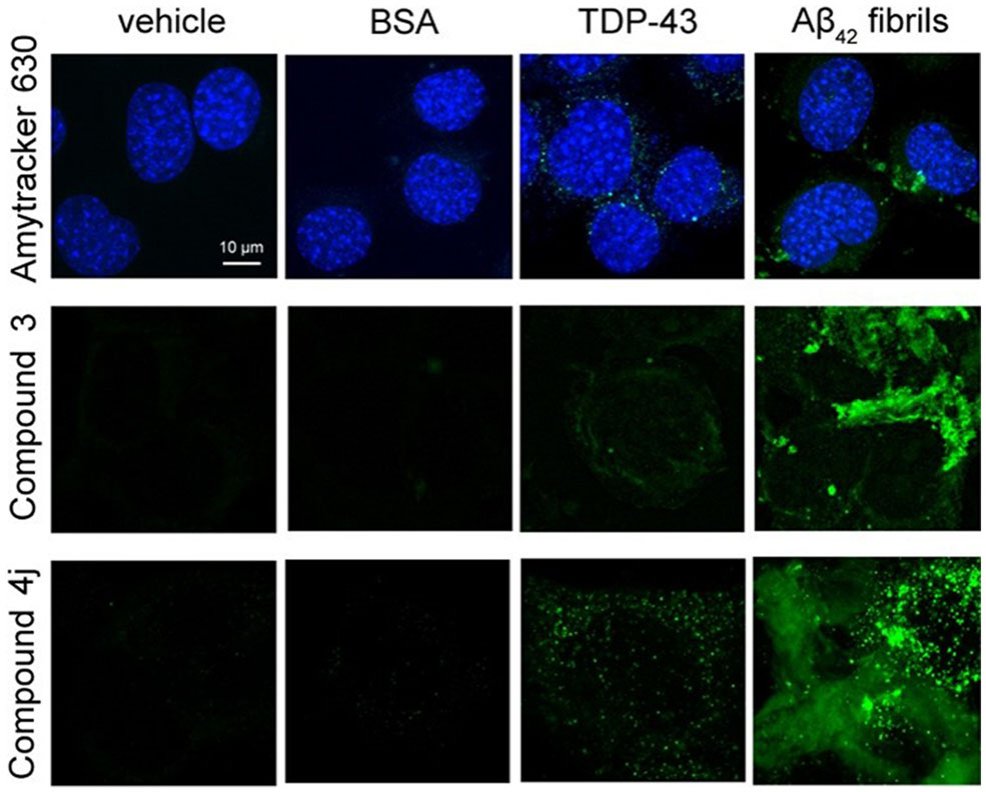
Cytoplasmic inclusions of TDP-43 do not stain with amyloid-diagnostic dyes able to cross biological membranes. Representative confocal microscopy images of NSC-34 cells (*n* = 3) transfected with vehicle, 0.15 μM BSA, 20 µg of pCI-neo plasmid expressing human TDP-43, or preformed Aβ_42_ fibrils at 2 µM monomer equivalents. Cells were then stained with Amytracker 630 (upper panels), Compound **3** (middle panels), and compound **4j** (lower panels). Green and blue fluorescence: amyloid-bound diagnostic dyes and DAPI-stained nuclei, respectively.

We used three specific amyloid-diagnostic dyes that have the ability to cross biological membranes and increase their fluorescence if bound to amyloid cytoplasmic inclusions. In particular, NSC-34 cells were stained with Amytracker 630 [46], with an aurone-derived molecule, referred to as compound **3** [47], and with a thiobarbituric acid-based chromene derivative called compound **4j** [48]. In previous studies *in vitro,* compounds **3** and **4j** were found to be able to discriminate between amyloid and amorphous aggregation/native protein forms by increasing the fluorescence signal [47,48]. In NSC-34 cells transfected with vehicle or soluble bovine serum albumin (BSA) as negative controls, we did not observe any fluorescence (Figure 2). In NSC-34 cells overexpressing TDP-43 (40 h after transfection), we observed a very low fluorescence in the cytoplasm with all three probes, suggesting either a substantially non-amyloid nature of TDP-43 inclusions or very few inclusions with amyloid structure (Figure 2). When NSC-34 cells were transfected with pre-formed fibrils of the 42-residue form of the amyloid β (Aβ_42_) peptide as a positive control, we observed a dramatic increase in the intracellular dye-derived fluorescence with all three probes, confirming the ability of the probes to detect specifically amyloid assemblies present in the cytoplasm (Figure 2).

### Cytoplasmic inclusions of TDP-43 are not enriched with cross-β structure

**Figure 3.**
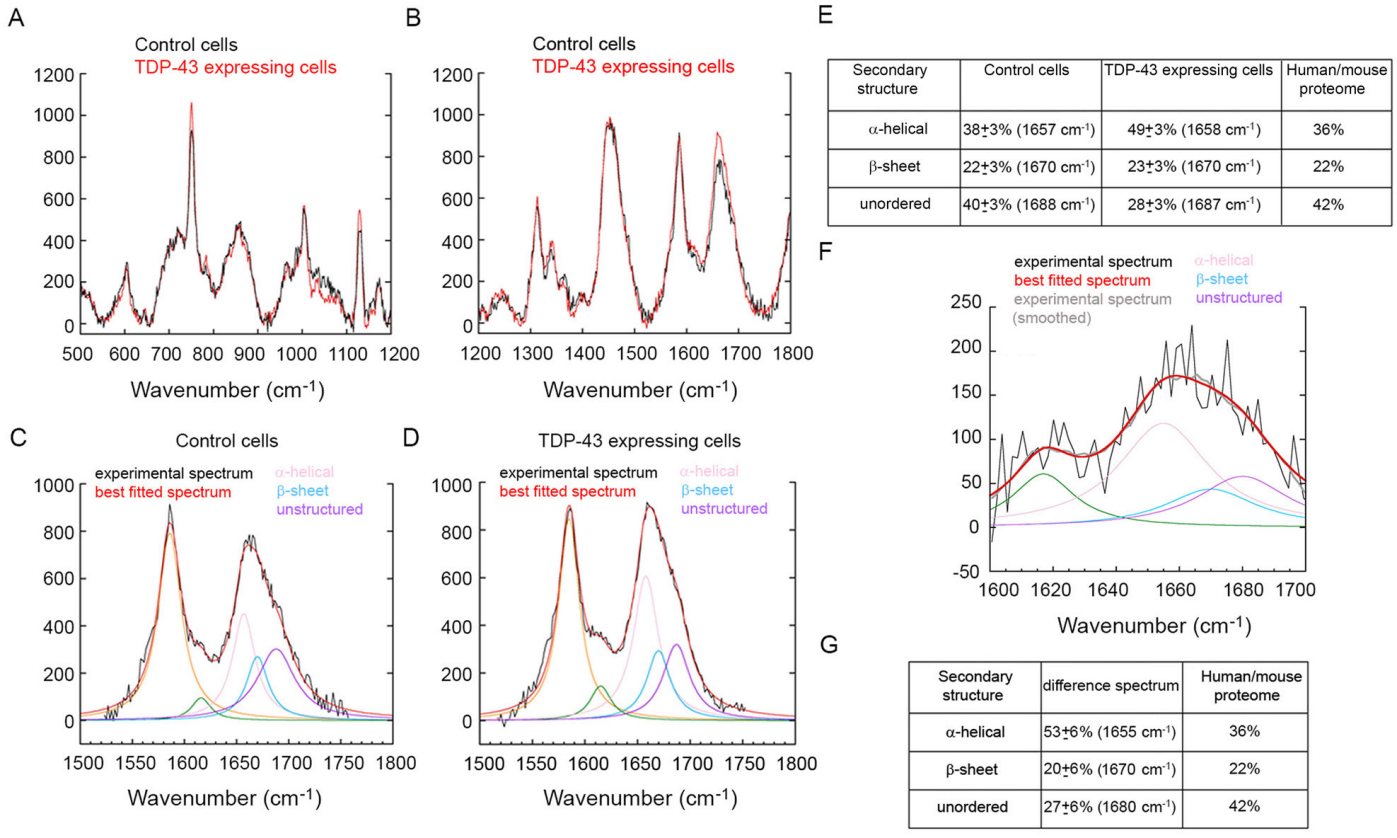
Cytoplasmic inclusions of TDP-43 do not have an enrichment of β-sheet structure. (A,B) Raman spectra (*n* = 15) of control cells (black) and TDP-43 expressing cells (red) in the 500–1200 cm^−1^ wavenumber range with excitation at 532 nm (A) and in the 1200–1800 cm^−1^ wavenumber range with excitation at 785 nm (B). (C,D) Magnification of the 1500–1800 cm^−1^ region (*n* = 15) containing the amide I band for control cells (C) and TDP-43 expressing cells (D), showing the experimental spectrum (black), the best fitted spectrum using a sum of five Lorentzian functions (red) and individual fitted components (indicated colors). (E) Quantification of secondary structure components for both control cells and TDP-43 expressing cells. The last column reports the data of the mammalian proteome [49,50]. (F) Difference spectrum obtained by subtracting the control cell spectrum from that of TDP-43 expressing cells: experimental (black), smoothed (grey), best fitted (red) spectrum, and individual fitted components (indicated colors). (G) Quantification of secondary structure components of the difference spectrum.

To explore the secondary structure of the TDP-43 cytoplasmic inclusions *in situ* in the NSC-34 cells, we used Raman microspectroscopy. In these experiments, human full-length TDP-43 was expressed as a protein C-terminally fused with the fluorescent tdTOMATO protein, to monitor with fluorescence the cytoplasmic areas enriched with TDP-43 inclusions within the NSC-34 cells and record Raman spectra in these areas. The Raman spectra of control cells and TDP-43 expressing cells feature significant differences in both the wavenumber range of 500–1200 cm^−1^ with excitation at 532 nm (Figure 3(A)) and 1200–1800 cm^−1^ with excitation at 785 nm (Figure 3(B)). A significant increase of band intensity in TDP-43 cells relative to control cells is observable at *ca.* 750, 1130, 1220–1260, 1340, 1610–1625, and 1630–1700 cm^−1^.

The first two bands are characteristic of the heme group of cytochrome c (cyt-c), as reported [52]. It is known that under apoptosis triggered by increased levels of Ca^2+^ and ROS (a common situation when aggregates are present including TDP-43 aggregates [34], cyt-c is released from the mitochondria into the cytoplasm [53,54], explaining why cyt-c bands increase in intensity in cytoplasmic areas enriched with TDP-43 inclusions (Figure 3(A)). The bands at 1610–1625 cm^−1^ and that at 1340 cm^−1^ are associated with aromatic groups [54] and are not of interest in the context of the present analysis.

The bands at *ca.* 1220–1260 and 1630–1700 cm^−1^ correspond to the amide III and amide I bands, respectively [52]. Both appear to be enhanced in intensity in TDP-43 enriched regions due to the presence of accumulated proteins (Figure 3(B)). The amide I band of each of the two spectra was fitted with a sum of Lorentzian functions, as detailed in the *Methods* section, and the amide I components emerging from the analysis (Figure 3(C,D)) were assigned to well-defined secondary structure types for the quantification of their amounts in the cellular areas of analysis (Figure 3(E)). The analysis of control cells indicates a distribution of the three main secondary types (α-helical, β-sheet, and unordered) compatible with that known in the mammalian proteome [49,50], within experimental error (Figure 3(E)). The analysis of TDP-43 expressing cells indicates a significant enrichment of α-helical structure and a decrease of unordered structure, whereas the β-sheet structure remains similar (Figure 3(E)). This indicates that cytoplasmic areas with accumulated TDP-43 do not have an enrichment of β-structured aggregates. As a positive control, we show the spectrum of cytoplasmic areas of neurons with fibrillar α-synuclein containing β-sheets obtained from the frontal cortex of a dementia with Lewy bodies case and measured using the same protocol and Raman configuration (Figure S1). The enrichment of α-helical structure in the cytoplasmic inclusions of TDP-43 expressing cells may have many explanations, such as structural rearrangement of TDP-43 in the aggregates (particularly the normally unstructured CTD), accumulation of chaperones in these areas (Hsp70s, Hsp90s, prefoldin, UPS have a high content of α-helices), accumulation of cyt-c (all-α protein), etc.

As a further inspection we also analyzed the amide I difference spectrum, obtained by subtracting the spectrum of control cells from that of TDP-43 expressing cells (Figure 3(F)). The difference spectrum is very noisy because the difference between the two spectra is small, albeit significant, and was therefore smoothed before the fitting analysis performed as described above. The relative quantities of the various secondary structure types obtained from the difference spectrum indicate enrichment and loss of α-helical and unordered structure, respectively, and an overall maintenance of β-sheet structure, in agreement with the results on the gross spectra (Figure 3(G)). Overall, a sharp peak around 1670 cm^−1^ arising from β-sheet structure is missing and does not emerge as an evident shoulder in the TDP-43 or difference spectra relative to the control spectrum.

To have an independent measurement of the secondary structure of the TDP-43 cytoplasmic inclusions *in situ*, we also used FTIR microspectroscopy, which has been successfully used for other systems to detect *in situ* the cross-β structure within amyloid deposits [55,56]. In these experiments, human full-length TDP-43 was expressed without the tdTOMATO protein. The FTIR spectra in the amide I region (1700–1600 cm^−1^) of control cells and TDP-43 expressing cells indicate the absence of an increased absorption in the 1630–1615 cm^−1^ range in the second spectrum relative to the first (Figures S2(A,B)), ruling out an enrichment of intermolecular cross-β structure in TDP-43 expressing cells. The enrichment of α-helical structure at the expense of unordered structure, detected with Raman microspectroscopy in the TDP-43 expressing cells, remains undetected with FTIR spectroscopy because the absorptions of the two secondary structure types overlap closely in the amide I band (Figures S2(A,B)). As a positive control, we show the spectrum obtained with the same procedure and technical apparatus of FTIR microspectroscopy as those used here of a cardiac tissue section of a human patient affected by light chain amyloidosis (AL) containing amyloid fibrils with cross-β structure of an immunoglobulin light chain (Figures S2(C,D)).

### Cytoplasmic inclusions of TDP-43 do not show a fibrillar morphology

**Figure 4.**
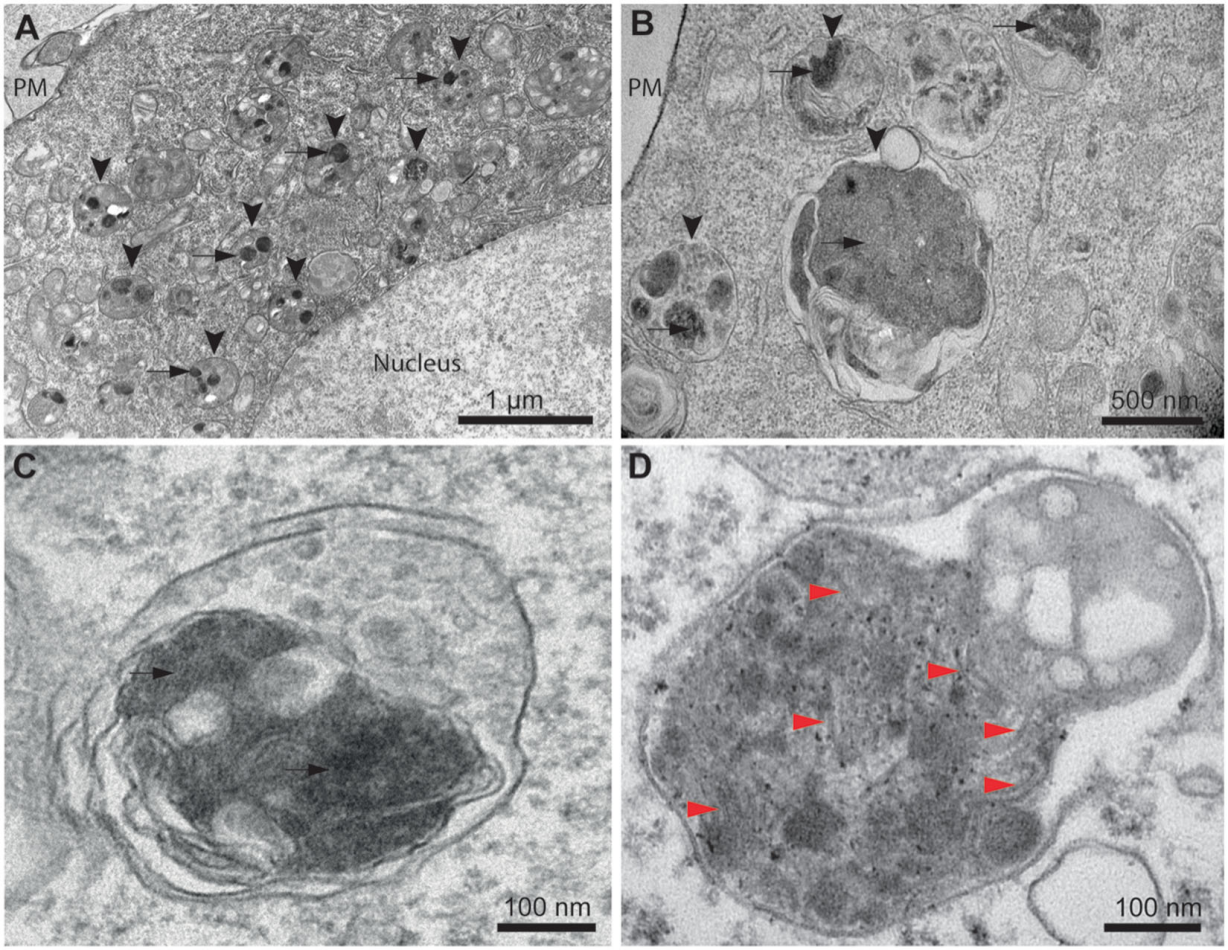
Cytoplasmic inclusions of TDP-43 imaged with TEM accumulate in lysosomes and show a non-fibrillar morphology. (A) Representative electron micrograph showing lysosomal accumulation (arrowheads) in NSC-34 cells expressing TDP-43 after 40 h from transfection. Note the round shaped electron-dense inclusions within lysosomes (arrows). Plasma membrane (PM) and nucleus are also indicated. (B) Higher magnification image showing lysosomes (arrowheads) engulfed with electron-dense material and putative TDP-43 inclusions (arrows). (C) Further enlargement of an image showing a lysosome with non-fibrillar dense inclusions (arrows). (D) Positive control with a high magnification image of a lysosome from a primary neuron of PS19 mice expressing P301S mutant human tau (gene *MAPT*), showing filamentous inclusions (red arrowheads).

Ultrastructural analysis of TDP-43 expressing cells using TEM (40 h after transfection) revealed that the cytoplasmic inclusions appear in most cases localized within the lysosomal compartments, indicating an active autophagy process (Figure 4(A), arrowheads). Round and electron-dense inclusions are present with sizes up to 0.50–1.50 µm in the absence of an evident fibrillar morphology (Figures 4(B,C), arrows). Close inspection of TDP-43 inclusions in magnified images revealed the presence of very few filaments (Figure 4(C)). These are rare and do not appear as straight and long fibrils typical of amyloid [21–23], nor the TDP-43 filaments observed in ALS/FTLD-U post-mortem specimens [10,14,26–30]. As a positive control, we used cultured neurons from transgenic PS19 mice expressing human P301S mutant tau and we acquired the electron micrograph of a lysosome, presenting numerous paired helical filaments (Figure 4(D), red arrowheads).

### *TDP-43 inclusions formed* in vitro *from pure TDP-43 do not have any hallmarks of amyloid*

To assess the intrinsic propensity of TDP-43 to form amyloid fibrils *in vitro* in a buffer solution mimicking physiological conditions, we purified human full-length native TDP-43 and diluted it from *ca.* 35 µM of its storing solution, in which it was soluble, to 5 µM in 20 mM acetate buffer, 150 mM NaCl, 5% (w/v) PEG8000, 2 mM TCEP, pH 5.0, to reach a final pH of 6.0, and incubated under shaking for 4 days at 25 °C.

**Figure 5.**
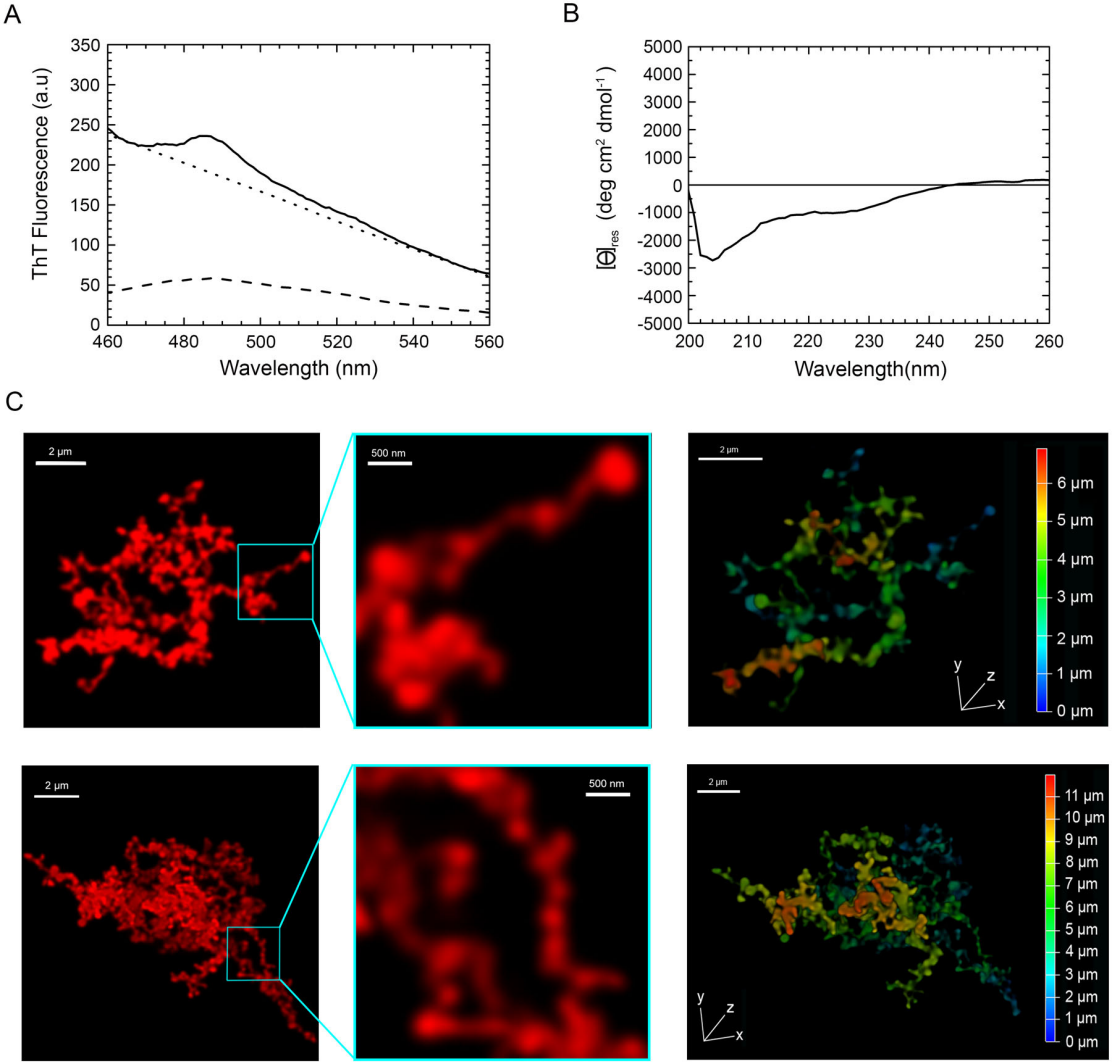
Tinctorial, structural and morphological analysis of TDP-43 aggregates formed *in vitro* from purified full-length TDP-43 show a non-amyloid nature. (A) ThT fluorescence spectrum in the presence of TDP-43 preincubated at a concentration of 5 µM, in 20 mM acetate buffer, 150 mM NaCl, 5% (w/v) PEG8000, 2 mM TCEP, pH 5.0 (Final pH 6.0), 25 °C, under agitation at 560 rpm for 4 days (solid line). The ThT-independent baseline deriving from aggregate-generated light scattering is shown (dotted line). The ThT fluorescence spectrum in the absence of protein (blank) is also shown (dashed line). (B) Far-UV CD spectrum of TDP-43 pre-incubated as described above. (C) Left: representative confocal microscopy images showing TMR-TDP-43 protein pre-incubated as described above. Center: higher magnifications of the aggregates shown from the boxed areas. Right: depth coding profiles along the *xyz*-axes of the 3D reconstruction of the same aggregates (different colors represent different planes along the *xyz* axes).

The ThT fluorescence spectrum in the absence of protein (blank) had a maximum at *ca.* 485 nm with a value of ca 50a.u. (Figure 5(A), dashed line). The ThT fluorescence spectrum in the presence of TDP-43 aggregates formed after 4 days was affected by a heavy wavelength-dependent and ThT-independent light scattering effect that increased the apparent fluorescence emission baseline (Figure 5(A), solid line). The spectrum maintained the typical peak at 485 nm, with an increase of ca. 50a.u. above the ‘light scattering baseline’ obtained with TDP-43 aggregates in the absence of ThT (Figure 5(A), dotted line), similar to that observed for free ThT. This is in contrast with the over 5-fold fluorescence increase expected for amyloid [57,58], ruling out the formation of amyloid species for TDP-43 upon these experimental conditions.

The far-UV circular dichroism (CD) spectrum of the TDP-43 aggregates formed after 4 days showed a prominent negative peak at *ca.* 204 nm and a shoulder around 222 nm, which reveals the presence of α-helix and random coil secondary structure, particularly the former (Figure 5(B)). The CD spectrum did not show any negative peak at 215–220 nm, typical of β-sheet structure; nor was a peak beyond 225 nm observed, as often found for large protein aggregates with β-sheet structure and producing differential absorption flattening (Figure 5(B)). This suggests the lack of a dominant β-sheet content and rules out the formation of an amyloid structure.

Confocal microscopy images of the same TDP-43 sample, but pre-labelled with the TMR fluorophore, revealed large aggregates of TDP-43 with no defined ordered morphology and a size of *ca* 10 µm (Figure 5(C), left panels). Moreover, the morphologies of individual aggregates diverged clearly from each other, underlining the absence of homogeneity during the aggregation process. A non-regular fibrillar morphology was apparent at high magnification with diameters below 200 nm (Figure 5(C), middle panels). Aggregates were further depth coded to allow a better characterization of their 3D morphology, revealing a depth along the z-axis (Figure 5(C), right panels, Supplementary Movie S3). To assess whether the large non-filamentous aggregates analyzed after 4 days reorganize into filamentous species upon prolonged incubation under the same conditions, we acquired confocal images of the same sample after 10 days, which is a time length sufficiently high for proteins to form amyloid fibrils when they have the propensity to form such assemblies [59–65]. In this case, we used STED super-resolution confocal microscopy to visualize a possible fibrillar texture. The images showed aggregates that were larger than those observed at 4 days, with the size of *ca* 20 µm, yet in the absence of a defined ordered fibrillar morphology (Figure S4).

Overall, since amyloid aggregates generally have an orderly and rigid fibrillar morphology, with the formation of cross-β structure that can induce an increase of ThT fluorescence emission by over *ca* 5-fold, our results suggest that TDP-43 aggregates formed *in vitro* under these conditions did not show typical amyloid characteristics. Our lab has previously identified these characteristics in many protein aggregates formed *in vitro* from at least seven different protein systems, therefore acting as positive controls of amyloid [59–65].

## Discussion

In this work, we have expressed human full-length TDP-43 and observed its self-assembly in the immortalized NSC-34 cell culture model, which recapitulates many of the characteristics of motor neurons and is the model cell line of election for ALS-related studies [18,40,66]. We have also incubated the same protein, purified in a native form using a previously published protocol [20], in a buffer close to physiological to induce its aggregation, which we observed for up 10 days. In both cases, the protein forms inclusions that are sufficiently large to be observed with conventional confocal microscopy. Both the intracellular inclusions and the isolated aggregates do not bind amyloid-diagnostic dyes or do so only weakly, with a very small increase in their fluorescence. An enrichment of β-sheet structure remained clearly undetected using both Raman and FTIR microspectroscopies for cytoplasmic inclusions and far-UV CD for *in vitro* self-assemblies. Using TEM and super-resolution STED microscopy, the cytoplasmic inclusions do not show any obvious fibrillar morphology, with a few fibrils appearing only occasionally within the inclusions.

The investigation of the TDP-43 cytoplasmic inclusions was carried out *in situ*. To this purpose, we used three amyloid-diagnostic dyes that have the ability to cross biological membranes and stain amyloid in the cytoplasm [46–48], Raman and FTIR microspectroscopies to detect possible β-sheet structure within the inclusions [52,55,56], and TEM that is normally used to reveal the typical 10–20 nm wide filaments within TDP-43 NCIs in post-mortem specimens [10,14,26–30,35]. The aggregates from pure full-length TDP-43 and assembled up to 10 days were formed from an initially native protein, unlike those analyzed in our previous report that was obtained from a protein initially unfolded in urea [67].

An important difference between the morphology of our cellular and *in vitro* assemblies and that detected in post-mortem NCIs is the absence of a clear filamentous texture in our two conditions of analysis. This may result from the shorter time scale of the aggregation process in our cellular and test-tube conditions, as opposed to the slow process occurring in pathology where more ordered filaments can form upon slow conversion of the early amorphous inclusions or be seeded by rarely and occasionally formed filaments that then propagate rapidly. Filaments were not observed, however, *in vitro* from pure TDP-43, even after 10 days of incubation, which is a time largely sufficient for purified proteins to form amyloid fibrils when they have the propensity to form such assemblies [59–65]. Similarly, filaments were not observed in cells upon plasmid transient transfection and TDP-43 overexpression after 40 h, which is a procedure that leads to amyloid fibril formation when expressing amyloidogenic proteins, such as α-synuclein and tau [68–70]. By contrast, in spite of this very low propensity to form filamentous structures *in cell* and in the test tube, TDP-43 exhibits a very high propensity to form solid-phase aggregates in both environments, under conditions of pH and temperature that do not otherwise promote aggregation of stable proteins.

Another important difference with the cryo-EM post-mortem filaments is the enrichment of α-helical structure at the expense of random coil structure. This probably results from the collapse of the CTD that is initially largely unfolded in the native protein but may present the characteristic α-helical enrichment of early-formed protein aggregates [71]. Later on, it is likely that these aggregated species convert in the recently determined double-spiral fold, but our secondary structure analysis carried out *in cell* and *in vitro* shows that this does not yet occur in the initial steps of TDP-43 aggregation.

One of the advantages of our experimental approach is the possibility to assess the intrinsic propensity of the TDP-43 protein to form any given inclusion type spontaneously, on a time scale that is otherwise sufficient for amyloid to form. Another advantage is offered by the opportunity to detect and analyze the early inclusions that may eventually lead to filaments, which is a step that escapes detection when analyzing only post-mortem specimens. Finally, our analysis has been carried out in the absence of TMEM106B amyloid fibrils that may mislead and confound the analysis of natural specimens *in vivo* (see below).

The identification of non-amyloid TDP-43 assemblies formed *in vitro*, in a non-cellular context, is not entirely novel. Early reports published in 2009–2014 showed that full-length TDP-43 can form filaments that were not able to bind ThT or Congo red [72], or induced a weak fluorescence increase of ThT or ThS [73,74], which was found to be much lower than the 5-fold increase normally found for amyloid fibrils [57,58]. Over the same time period, full-length TDP-43 was found to form large 40–60 nm spheroidal or ring-shaped aggregates enriched with α-helical structure and unable to bind ThT [75]. A later report described the conversion of full-length TDP-43 fused to the yellow fluorescent protein (YFP) into aggregates that had an irregular, tuft-like, flocculant morphology incapable of binding ThT [31], which was later confirmed by a protein devoid of large tags [76]. Another very recent report showed that full-length TDP-43, purified in a urea-denatured state and dialyzed against a physiological buffer, was also capable of forming filaments without cross-β structure, as indicated with far-UV CD and conventional FTIR spectroscopy, and without ThT or Congo red binding [67]. Similarly, recombinant full-length TDP-43 was converted *in vitro* into aggregates that presented both fibrils and amorphous aggregates with, again, a weak ThT fluorescence increase [77]. Finally, incubation of initially native TDP-43 into conditions close to physiological, in the absence of thermal or acidic stress, led to large oligomers that clustered together into amorphous species and enriched with α-helical structure [78].

Histopathologists also imaged, in post-mortem ALS and FTLD cases, TDP-43 NCIs in the absence of ThT/ThS or Congo red binding [31–33,35]. The absence of a ThT positivity or even fibrillar structure has also been reported recently on post-mortem inclusions of TDP-43 obtained with the SarkoSpin method [35,38]. In spite of these observations, other reports have called the non-amyloid nature of the TDP-43 into question. A report described ThS-positive TDP-43 inclusions in ALS patients, although the ThS positivity was limited to a small fraction of skein-like inclusions of the spinal cord and it was altogether absent in round spinal cord inclusions and in the brain, independently of the NCI morphology [28]. A diffuse ThS positivity was found in all the analyzed TDP-43 inclusions of ALS spinal cords and FTLD-U brains after a heavy chemical treatment [36]. Furthermore, the CTD of TDP-43, as well as short peptides from its sequence or from that of the RRM2 domain, have been converted into amyloid-like fibrils *in vitro* under appropriate conditions [79–85]. Such observations caused a paradigm shift and led to the assumption, within a large fraction of the scientific community, that even full length TDP-43 may have an amyloid structure in the inclusions.

Much of this debate has been resolved by the recent cryo-EM structural elucidation of the TDP-43 filaments from two distinct patients, both with a history of ALS and FTLD-U [30]. Both structures share a very peculiar and unprecedented double-spiral fold in which (i) the β-strands perpendicular to the fibril axis are very short and alternate with long stretches of turns, (ii) the very narrow β-sheets running along the fibril axis do not stack, and do not form the cross-β structure typical of amyloid, and (iii) the flat surface on the filament does not contain the deep and nonpolar grooves required to bind ThT/ThS or related compounds [30]. The same report revealed a cryo-EM filament structure different from that previously published with cryo-EM on filaments formed *in vitro* with the TDP-43 CTD or even shorter CTD peptides, that have by contrast amyloid characteristics [78–84]. In addition, the discovery that TDP-43 inclusions often colocalize with amyloid fibrils by the TMEM106B CTD in ALS and FTLD-U patients [37–39], suggests that the occasional ThT/ThS observed in the human specimen may arise from such structures rather than TDP-43 assemblies.

We also believe that this debate can be reconciled following the knowledge accumulated on the fundamentals of amyloid fibril formation over the past  25 years. Small peptides and protein fragments are well known to have a high propensity to form amyloid-like fibrils through a mechanism and pathway independent of the corresponding full-length proteins [21]. Large proteins are less prone to form amyloid in pathology and a sequence length of *ca.* 250–350 residues has been proposed as a threshold beyond which amyloid formation is rarely observed in pathology [86]. In this regard, it is not surprising that most reports on the aggregation of CTD (*ca*. 150 residues) and full-length TDP-43 (414 residues) have shown the formation of amyloid and non-amyloid assemblies, respectively. The finding by Bigio et al. of a marked ThS positivity in ALS and FTLD-U specimens probably originated from the chemically harsh treatment of the tissue sections, based on the sequential use of potent oxidants, reductants, acids, and bases [36], all known to induce conformational changes within proteins and even hydrolyze their peptide bonds. It cannot be excluded that the biological inclusions undergo a substantial structural reorganization and even fragmentation following this treatment, with a higher propensity to form amyloid. Alternatively, TMEM106B fibrils might have been detected in this case. In all other studies performed *in vitro* [31,34,67,72–78], *in cell* (present work), and *in vivo* inclusions [28,30–33,35,38], a clear amyloid signature remains undetected.

## Conclusions

**Figure 6.**
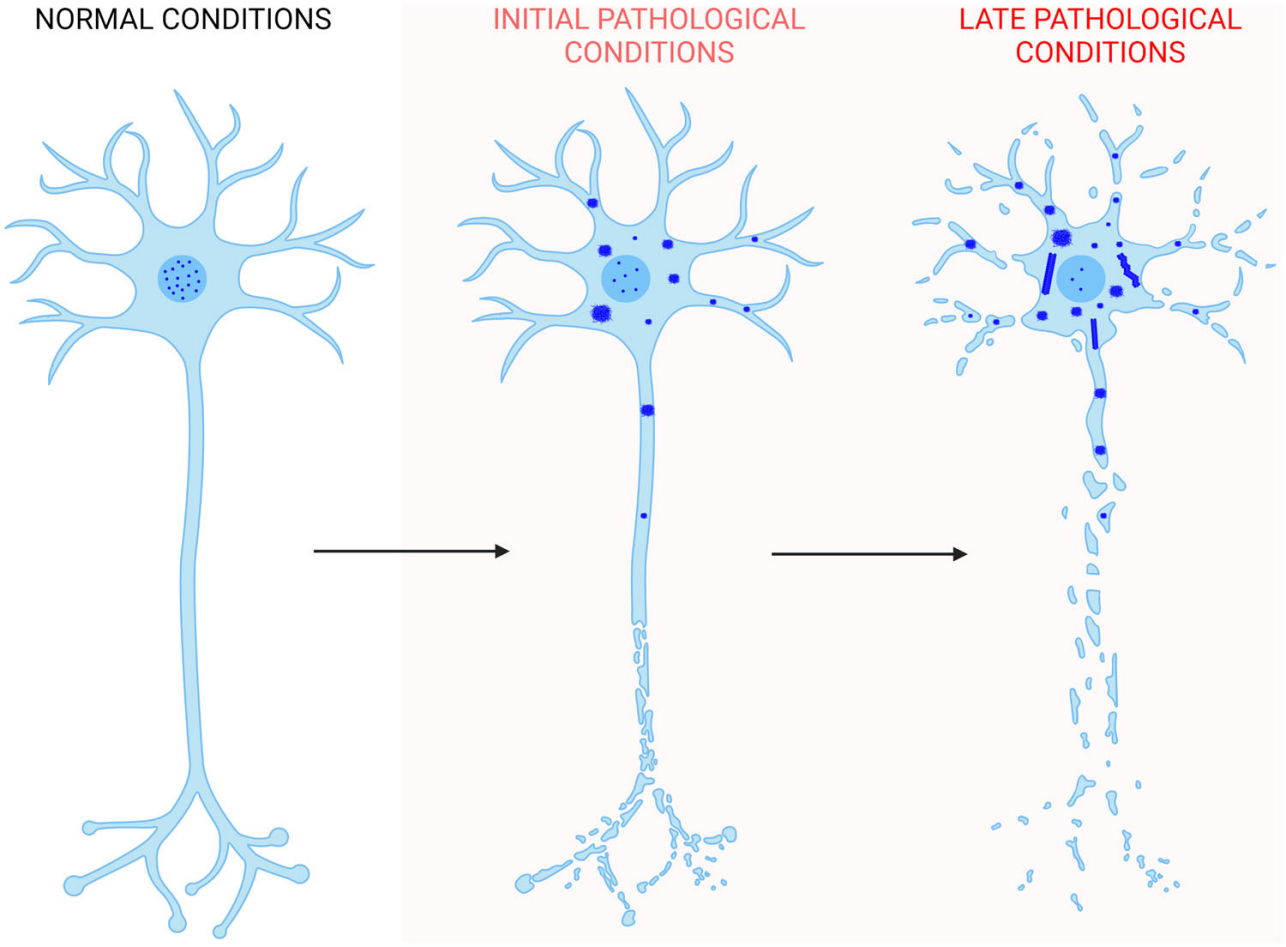
Scheme of TDP-43 inclusion formation. Self-assembly of full-length TDP-43 (blue) begins with the nucleus-to-cytoplasm mislocalization of the protein. In the cytoplasm it forms non-amyloid inclusions of different sizes. Later on, filaments distinct from the classical amyloid fibrils may or may not form. In the first case, they can either form directly from a structural reorganization of the initial inclusions, or from nuclei derived slowly, rarely, and independently of them. In the latter scenario, filaments would then rapidly grow from TDP-43 reservoirs provided by the initial non-filamentous inclusions. The process also involves phase separation within stress granules and TMEM106B CTD amyloid fibril formation, which have been neglected in the figure for simplicity (Created with BioRender.com).

The data presented here show the absence of a clear amyloid signature in both the TDP-43 inclusions formed in NSC-34 cells following overexpression of the protein and in the TDP-43 aggregates formed *in vitro* from the purified native protein under conditions close to physiological. In both cases, the inclusions do not bind to ThT or derived fluorophores specific for amyloid, do not possess a significant β-sheet structure, and do not appear fibrillar in morphology. We, therefore, propose that self-assembly of TDP-43 starts with the mislocalization of the protein from the nucleus to the cytoplasm [1–6], where it forms non-amyloid inclusions of different sizes in which the largest inclusions are responsible for neuron dysfunction, as we recently showed [87]. Later on, filaments without amyloid diagnostic dye binding and without the β-sheet stacking on the equatorial axis, typical of amyloid fibrils, may or may not arise from the inclusions, as shown by the presence [30] or absence [35,38] of such structures in post-mortem neuronal extracts (Figure 6). Such structures may originate from the conversion of the initial non-fibrillar inclusions or from nuclei derived slowly, rarely, and independently of them. In the latest scenario, filaments would then rapidly grow from TDP-43 reservoirs provided by the initial non-amyloid inclusions.

## Supplementary Material

Supplemental Material

Supplemental Material

## Data Availability

The data that support the findings of this study are available on request from the corresponding author.
