# Supplementary material for: An in situ and in vitro investigation of cytoplasmic TDP-43 inclusions reveals the absence of a clear amyloid signature
Source: Ann Med. 2022 Dec 10;55(1):72–88. doi: 10.1080/07853890.2022.2148734 (PMC9746631; doi:10.1080/07853890.2022.2148734)
Supplement: Supplemental Material [file IANN_A_2148734_SM2675.docx]

**Supplementary figures and legends**

**
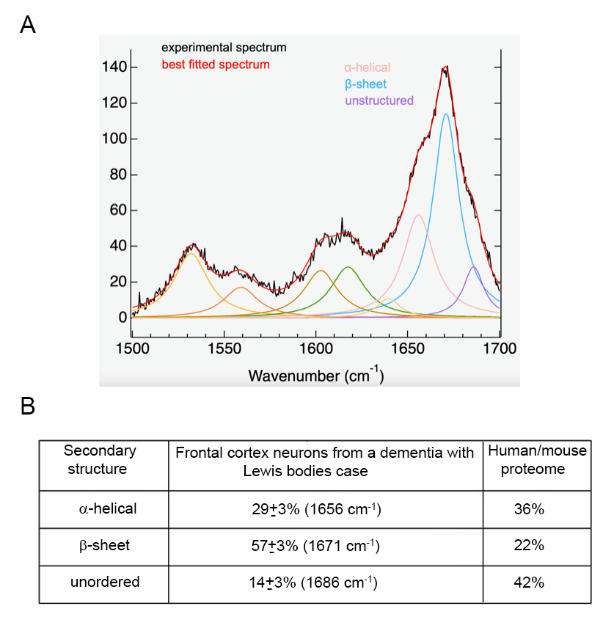
**

**Figure S1. Positive control of cytoplasmic inclusions enriched with β-sheet structure. (A)** Raman spectrum (n=5) of cytoplasmic inclusions of neurons containing fibrillar α-synuclein, with known enrichment of β-sheet structure, from brain tissue-derived samples obtained from the frontal cortex of a dementia with Lewy bodies case. The spectrum (excitation at 785 nm) shows the characteristic amide I band in the 1500-1700 cm^-1^ region. The figure shows the experimental spectrum (black), the best fitted spectrum using a sum of eight Lorentzian functions (red) and individual fitted components (indicated colors). **(B)** Quantification of secondary structure components of the spectrum showed in panel A. The last column reports the data of the mammalian proteome^49,50^ Readapted from^81^ under a Creative Commons Attribution 3.0 Unported Licence.

**
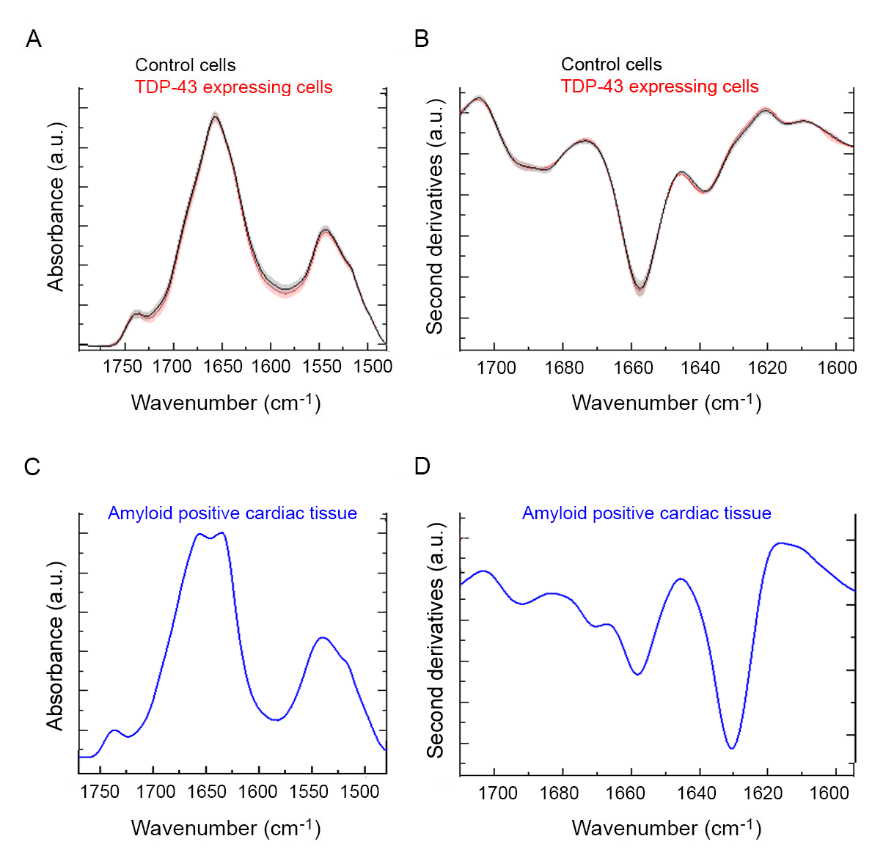
**

**Figure S2. FTIR microspectroscopy of control cells, TDP-43 expressing cells and of a positive control specimen containing amyloid fibrils.** (**A**) Absorption spectra in the amide I and amide II spectral ranges for control cells (black) and TDP-43 expressing cells (red). Both bands are sensitive to the protein secondary structures and appear to be superimposable in this spectral region. (**B**) Second derivatives in the amide I range of the absorption spectra reported in (A), showing two main components at around 1659 cm^-1^ (assigned to α-helical and random coil structures) and around 1639 cm^-1^ (assigned to β-sheet structures). No spectral changes in the 1630-1615 cm^-1^ region have been detected, indicating the absence of an increased intermolecular β-sheet structure in TDP-43 expressing cells, as instead typically observed for amyloid aggregates. The mean (solid lines) and standard deviation (shadows) from 40 measured spectra are reported for both control cells (black) and TDP-43 expressing cells (red). **(C)** Positive control showing the absorption spectrum in the amide I and amide II spectral ranges for a cardiac tissue section from the HT1 patient affected by light chain amyloidosis (AL) and containing large amounts of cross-β containing amyloid fibrils^55^. **(D)** Second derivative in the amide I range of the absorption spectrum reported in (C). The absorption and second derivative spectra display a minor component at around 1657 cm^-1^ (assigned to α-helical and random coil structures) and a main component at around 1630 cm^-1^ (assigned to intermolecular β-sheet structure). The latter spectral component was not observed in the amyloid-negative heart samples^55^. Readapted from^55^ under a Creative Commons Attribution 3.0 Unported Licence.


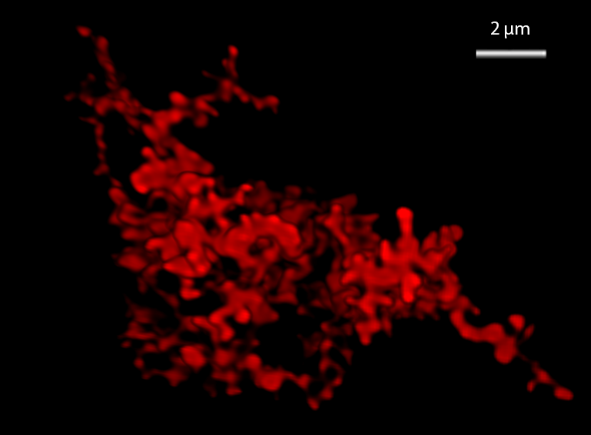


**Figure S3. An overall dynamic view of a TMR-TDP-43 aggregate in the 3D space.** The movie refers to the aggregate shown in Figure 5C (bottom left panel).

**
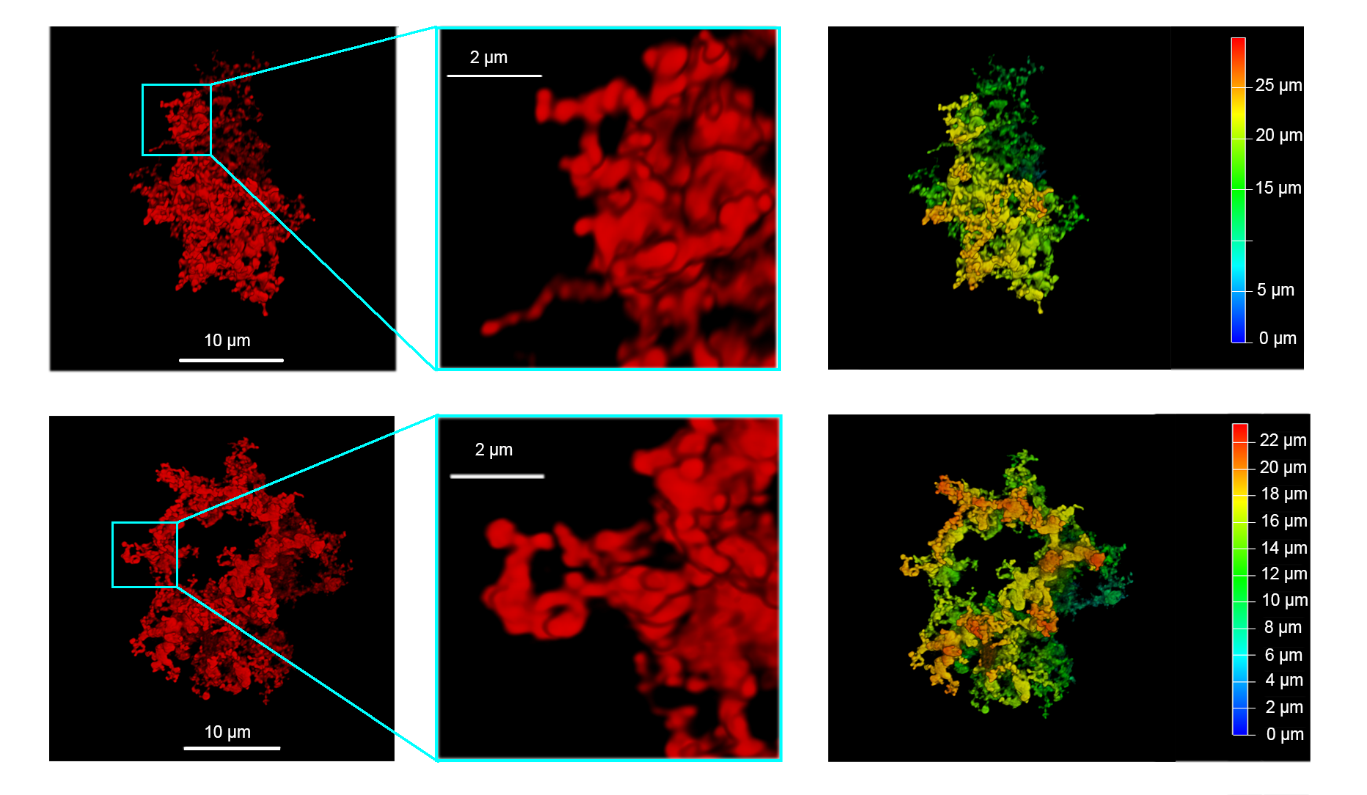
**

**Figure S4. Morphological analysis of TDP-43 aggregates incubated *in vitro* for 10 days**. Left: representative STED microscopy images showing TMR-TDP-43 protein pre-incubated at a concentration of 5 µM, in 20 mM acetate buffer, 150 mM NaCl, 5% (w/v) PEG8000, 2 mM TCEP, pH 5.0 (Final pH 6.0), 25 °C, under agitation at 560 rpm for 10 days. Center: higher magnifications of the aggregates shown from the boxed areas. Right: depth coding profiles along the xyz-axes of the 3D reconstruction of the same aggregates (different colors represent different planes along the xyz axes).

**Supplementary references**

Candelise N, Schmitz M, Llorens F, Villar-Piqué A, Cramm M, Thom T, da Silva Correia SM, da Cunha JEG, Möbius W, Outeiro TF, Álvarez VG, Banchelli M, D'Andrea C, de Angelis M, Zafar S, Rabano A, Matteini P, Zerr I. Seeding variability of different alpha synuclein strains in synucleinopathies. *Ann Neurol*. 2019; 85: 691-703.
